# The Phylogenetic Relationship Revealed Three New Wood-Inhabiting Fungal Species From Genus *Trechispora*

**DOI:** 10.3389/fmicb.2021.650195

**Published:** 2021-03-17

**Authors:** Wei Zhao, Chang-Lin Zhao

**Affiliations:** ^1^Key Laboratory for Forest Resources Conservation and Utilization in the Southwest Mountains of China, Ministry of Education, Southwest Forestry University, Kunming, China; ^2^College of Biodiversity Conservation, Southwest Forestry University, Kunming, China

**Keywords:** Hydnodontaceae, phylogeny, taxonomy, wood-inhabiting fungi, Yunnan Province

## Abstract

Wood-inhabiting fungi play a significant role in wood degradation and the cycle of matter in the ecological system. In the present study, three new wood-inhabiting fungal species, *Trechispora bambusicola*, *Trechispora fimbriata*, and *Trechispora fissurata* spp. nov., are nested in *Trechispora*, which are proposed based on a combination of morphological features and molecular evidence. Sequences of internal transcribed spacer (ITS) and large subunit (nLSU) regions of the studied samples were generated, and the phylogenetic analyses were performed with maximum likelihood, maximum parsimony, and Bayesian inference methods. The phylogenetic analyses inferred from ITS showed that *T. bambusicola* was sister to *Trechispora stevensonii*, *T. fimbriata* grouped with *Trechispora nivea*, and *T. fissurata* grouped with *Trechispora echinospora*. The phylogenetic tree based on ITS + nLSU sequences demonstrated that *T. bambusicola* formed a single lineage and then grouped with *Trechispora rigida* and *T. stevensonii*. *T. fimbriata* was sister to *T. nivea*. *T. fissurata* grouped with *Trechispora thelephora*.

## Introduction

*Trechispora* P. Karst. (Hydnodontaceae, Trechisporales) was typified with *Trechispora onusta* P. Karst. (Karsten, 1890). It is characterized by the resupinate to effused basidiomata with smooth to hydnoid to poroid hymenophore, a monomitic or dimitic hyphal structure with clamped generative hyphae having typical ampullaceous septa, and short cylindric basidia and smooth to verrucose or aculeate basidiospores (Karsten, 1890; Bernicchia and Gorjón, 2010). About 49 species are currently known in *Trechispora* worldwide (Liberta, 1966, 1973; Larsson, 1994, 1995, 1996; Ryvarden, 2002; Trichiès and Schultheis, 2002; Miettinen and Larsson, 2006; Ordynets et al., 2015; Xu et al., 2019) and Index Fungorum^1^ and MycoBank^2^.

Larsson (2007) addressed the classification of corticioid fungi and revealed that *Trechispora farinacea* (Pers.) Liberta grouped with *Trechispora hymenocystis* (Berk. and Broome) K.H. Larss., in which both species nested within the family Hydnodontaceae Jülich. Based on the large subunit nuclear ribosomal RNA gene (nLSU) datasets, Albee-Scott and Kropp (2010) supported to transfer *Hydnodon thelephorus* (Lév.) Banker to *Trechispora* as *Trechispora thelephora* (Lév.) Ryvarden. The order Trechisporales was studied employing the internal transcribed spacer (ITS) and nLSU regions, in which it suggested that *Porpomyces* Jülich, *Sistotremastrum* J. Erikss., *Subulicystidium* Parmasto, and *Trechispora* belonged to a highly supported clade and *Trechispora* belongs to Hydnodontaceae and was closely related to *Brevicellicium* K.H. Larss. and Hjortstam (Telleria et al., 2013). A phylogenetic study of *Trechispora* was addressed and demonstrated that *Trechispora cyatheae* Ordynets, Langer and K.H. Larss. and *Trechispora echinocristallina* Ordynets, Langer and K.H. Larss. clustered into *Trechispora* as new members, inferred from the combined data of the ITS and LSU datasets (Ordynets et al., 2015). The phylogeny of Trechisporales was inferred from a combined dataset of ITS-nLSU sequences and showed that *Porpomyces*, *Scytinopogon* Singer, and *Trechispora* grouped together and nested within family Hydnodontaceae (Liu et al., 2019). Phylogram generated from analysis of ITS sequence dataset of *Trechispora* showed that *Trechispora echinospora* Telleria was sister to the clade formed by *Trechispora araneosa* (Hohn. and Litsch.) K.H. Larss., *T. farinacea*, *T. hymenocystis*, and *Trechispora mollusca* (Pers.) Liberta with a low support (Phookamsak et al., 2019). The ITS + nLSU dataset comprised 22 species and revealed that *Trechispora yunnanensis* C.L. Zhao formed a monophyletic lineage within *Trechispora* and was closely related to *Trechispora byssinella* (Bourdot) Liberta and *Trechispora laevis* K.H. Larss. (Xu et al., 2019).

During the studies on wood-inhabiting fungi in southern China, three species of *Trechispora* could not be assigned to any described species. Obtaining sequences from the new taxa, the authors examine taxonomy and phylogeny of three new species within the genus *Trechispora*, based on the ITS and nLSU sequences.

## Materials and Methods

### Morphology

The studied specimens are deposited at the herbarium of Southwest Forestry University (SWFC), Kunming, Yunnan Province, China. Macromorphological descriptions were based on field notes. Color terms follow Petersen (1996). Micromorphological data were obtained from the dried specimens and observed under a light microscope following Dai (2012). The following abbreviations were used: KOH = 5% potassium hydroxide, CB = Cotton Blue, CB− = acyanophilous, IKI = Melzer’s reagent, IKI− = both inamyloid and indextrinoid, L = mean spore length (arithmetic average for all spores), W = mean spore width (arithmetic average for all spores), Q = variation in the L/W ratios between the studied specimens, n (a/b) = number of spores (a) measured from given number (b) of specimens, spore measurements do not include ornamentation.

### Molecular Phylogeny

Cetyltrimethylammonium bromide (CTAB) rapid plant genome extraction kit-DN14 (Aidlab Biotechnologies Co., Ltd., Beijing, China) was used to obtain genomic deoxyribonucleic acid (DNA) from dried specimens, according to the manufacturer’s instructions following Zhao and Wu (2017). ITS region was amplified with primer pair ITS5 and ITS4 (White et al., 1990). Nuclear LSU region was amplified with primer pair LR0R and LR7^3^. The polymerase chain reaction (PCR) procedures for ITS and nLSU following Zhao and Wu (2017). The PCR products were purified and directly sequenced at Kunming Tsingke Biological Technology Limited Company, Kunming, Yunnan Province, China. All newly generated sequences were deposited at GenBank (Table 1).

**TABLE 1:** List of species, specimens, and GenBank accession numbers of sequences used in this study.

Species name	Sample no.	GenBank accession no.		References
		ITS	nLSU	
*Fibrodontia alba*	TNMF 24944	KC928274	KC928275	Yurchenko and Wu (2014)
*Fibrodontia gossypina*	GEL 5042	DQ249274	AY646100	Unpublished
*Trechispora araneosa*	KHL 8570	AF347084	AF347084	Larsson et al. (2004)
*Trechispora bambusicola*	CLZhao 3302	MW544021	MW520171	This study
*Trechispora bambusicola*	CLZhao 3305	MW544022	MW520172	This study
*Trechispora bispora*	CBS 142.63	MH858241	MH869842	Vu et al. (2019)
*Trechispora byssinella*	UC 2023068	KP814481	–	Unpublished
*Trechispora cohaerens*	TU 110332	UDB008249	–	Ordynets et al. (2015)
*Trechispora cohaerens*	TU 115568	UDB016421	–	Ordynets et al. (2015)
*Trechispora confinis*	KHL 11064	AF347081	AF347081	Larsson et al. (2004)
*Trechispora cyatheae*	FR-0219442	UDB024014	UDB024014	Ordynets et al. (2015)
*Trechispora cyatheae*	FR-0219443	UDB024015	UDB024015	Ordynets et al. (2015)
*Trechispora echinocristallina*	FR-0219445	UDB024018	UDB024018	Ordynets et al. (2015)
*Trechispora echinocristallina*	FR-0219448	UDB024022	UDB024022	Ordynets et al. (2015)
*Trechispora echinospora*	E11/37-03	JX392845	JX392846	Telleria et al. (2013)
*Trechispora echinospora*	E09/60-06	JX392847	JX392848	Telleria et al. (2013)
*Trechispora echinospora*	E11/37-05	–	JX392849	Telleria et al. (2013)
*Trechispora farinacea*	KHL 8451	AF347082	AF347082	Unpublished
*Trechispora farinacea*	KHL 8793	AF347089	AF347089	Larsson et al. (2004)
*Trechispora fissurata*	CLZhao 995	MW544026	MW520176	This study
*Trechispora fissurata*	CLZhao 4571	MW544027	MW520177	This study
*Trechispora fimbriata*	CLZhao 4154	MW544023	MW520173	This study
*Trechispora fimbriata*	CLZhao 7969	MW544024	MW520174	This study
*Trechispora fimbriata*	CLZhao 9006	MW544025	MW520175	This study
*Trechispora hymenocystis*	KHL 8795	AF347090	AF347090	Unpublished
*Trechispora hymenocystis*	TL 11112	UDB000778	UDB000778	Ordynets et al. (2015)
*Trechispora incisa*	EH 24/98	AF347085	–	Unpublished
*Trechispora kavinioides*	KGN 981002	AF347086	AF347086	Larsson et al. (2004)
*Trechispora laevis*	TU 115551	UDB016468	–	Ordynets et al. (2015)
*Trechispora mollusca*	DLL 2010-077	JQ673209	–	Ordynets et al. (2015)
*Trechispora mollusca*	DLL 2011-186	KJ140681	–	Ordynets et al. (2015)
*Trechispora nivea*	MA-Fungi 76238	JX392824	JX392825	Telleria et al. (2013)
*Trechispora nivea*	MA-Fungi 76257	JX392826	JX392827	Telleria et al. (2013)
*Trechispora nivea*	MA-Fungi 82480	JX392829	JX392830	Telleria et al. (2013)
*Trechispora nivea*	MA-Fungi 74044	JX392832	JX392833	Telleria et al. (2013)
*Trechispora regularis*	KHL 10881	AF347087	AF347087	Larsson et al. (2004)
*Trechispora rigida*	URM 85754	–	MH279999	Unpublished
*Trechispora stevensonii*	MA-Fungi 70669	JX392841	JX392842	Telleria et al. (2013)
*Trechispora stevensonii*	HJM 18087	–	MH290761	Unpublished
*Trechispora stevensonii*	KHL 14654	–	MH290762	Unpublished
*Trechispora stevensonii*	TU 115499	UDB016467	UDB016467	Ordynets et al. (2015)
*Trechispora stellulata*	UC 2022880	KP814437	–	Unpublished
*Trechispora stellulata*	UC 2023099	KP814451	–	Unpublished
*Trechispora subsphaerospora*	KHL 8511	AF347080	AF347080	Larsson et al. (2004)
*Trechispora thelephora*	URM 85757	–	MH280001	Unpublished
*Trechispora thelephora*	URM 85758	–	MH280002	Unpublished
*Trechispora yunnanensis*	CLZhao 210	MN654918	MN654921	Xu et al. (2019)
*Trechispora yunnanensis*	CLZhao 214	MN654919	MN654922	Xu et al. (2019)
*Trechispora yunnanensis*	CLZhao 215	MN654920	MN654923	Xu et al. (2019)

Sequencher 4.6 (GeneCodes, Ann Arbor, United States) was used to edit the DNA sequence. Sequences were aligned in MAFFT 7^4^ using the “G-INS-I” strategy and manually adjusted in BioEdit (Hall, 1999). The sequence alignment was deposited in TreeBase (submission ID 25879). Sequences of *Fibrodontia alba* Yurchenko and Sheng H. Wu and *Fibrodontia gossypina* Parmasto retrieved from GenBank were used as an outgroup in the ITS + nLSU analyses by following Ordynets et al. (2015).

Maximum parsimony (MP) analyses were applied to the ITS + nLSU dataset sequences. Approaches to phylogenetic analysis followed Zhao and Wu (2017), and the tree construction procedure was performed in PAUP^∗^ version 4.0b10 (Swofford, 2002). All characters were equally weighted and gaps were treated as missing data. Trees were inferred using the heuristic search option with tree-bisection reconnection (TBR) branch swapping and 1000 random sequence additions. Max-trees were set to 5000, branches of zero length were collapsed, and all parsimonious trees were saved. Clade robustness was assessed using a bootstrap (BT) analysis with 1000 replicates (Felsenstein, 1985). Descriptive tree statistics tree length (TL), consistency index (CI), retention index (RI), rescaled consistency index (RC), and homoplasy index (HI) were calculated for each Maximum Parsimonious Tree generated. Datamatrix was also analyzed using maximum likelihood (ML) approach with RAxML-HPC2 through the Cipres Science Gateway with GTR + I + G molecular evolution model^5^ (Miller et al., 2009). Branch support (BS) for ML analysis was determined by 1000 BT replicates.

MrModeltest 2.3 (Nylander, 2004) was used to determine the best-fit evolution model (GTR + I + G) for each data set for Bayesian inference (BI) of the phylogeny. BI was calculated with MrBayes 3.1.2 (Ronquist and Huelsenbeck, 2003). Four Markov chains were run for two runs from random starting trees for 1 million generations and trees were sampled every 100 generations; the first one-fourth of generations were discarded as burn-in. A majority rule consensus tree of all remaining trees was calculated. Branches were considered as significantly supported if they received ML BT values > 75%, MP BT values > 75%, or Bayesian posterior probabilities (PP) > 0.95.

## Results

### Molecular Phylogeny

In the ITS dataset, the sequences from 43 fungal specimens representing 25 species were included. The dataset had an aligned length of 1034 characters, of which 521 characters are constant, 86 are variable and parsimony-uninformative, and 427 are parsimony-informative. MP analysis yielded 26 equally parsimonious trees (TL = 2048, CI = 0.4561, HI = 0.5439, RI = 0.6174, RC = 0.2816). Best model for the ITS dataset estimated and applied in the Bayesian analysis: GTR + I + G, lset nst = 6, rates = invgamma; prset statefreqpr = dirichlet (1,1,1,1). Bayesian analysis and ML analysis resulted in a similar topology to MP analysis, with an average standard deviation of split frequencies = 0.009985 (BI).

**FIGURE 1:**
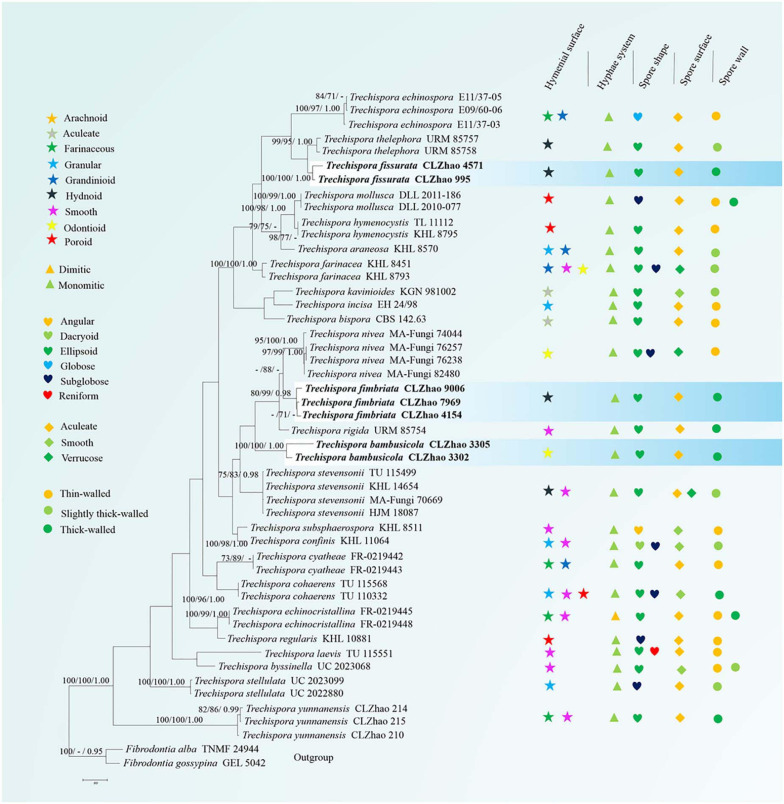
Maximum Parsimony strict consensus tree illustrating the phylogeny of three new species and related species in *Trechispora* based on ITS sequences. Branches are labeled with maximum likelihood bootstrap values > 70%, parsimony bootstrap proportion values > 50%, and Bayesian posterior probabilities > 0.95, respectively.

The phylogeny (Figure 1) inferred from ITS sequences showed that *Trechispora bambusicola* was sister to *Trechispora stevensonii* (Berk. and Broome) K.H. Larss, and *Trechispora fimbriata* grouped with *Trechispora nivea*. *T. fissurata* grouped with *T. echinospora* Telleria, M. Dueñas, I. Melo, and M.P. Martín.

In the ITS + nLSU dataset, it included sequences from 49 fungal specimens representing 27 species. The dataset had an aligned length of 2256 characters, of which 1387 characters are constant, 188 are variable and parsimony-uninformative, and 681 are parsimony-informative. MP analysis yielded 100 equally parsimonious trees (TL = 2811, CI = 0.4963, HI = 0.5037, RI = 0.6409, RC = 0.3180). Best model for the ITS dataset estimated and applied in the Bayesian analysis: GTR + I + G, lset nst = 6, rates = invgamma; prset statefreqpr = dirichlet (1,1,1,1). Bayesian analysis and ML analysis resulted in a similar topology to MP analysis, with an average standard deviation of split frequencies = 0.009991 (BI).

**FIGURE 2:**
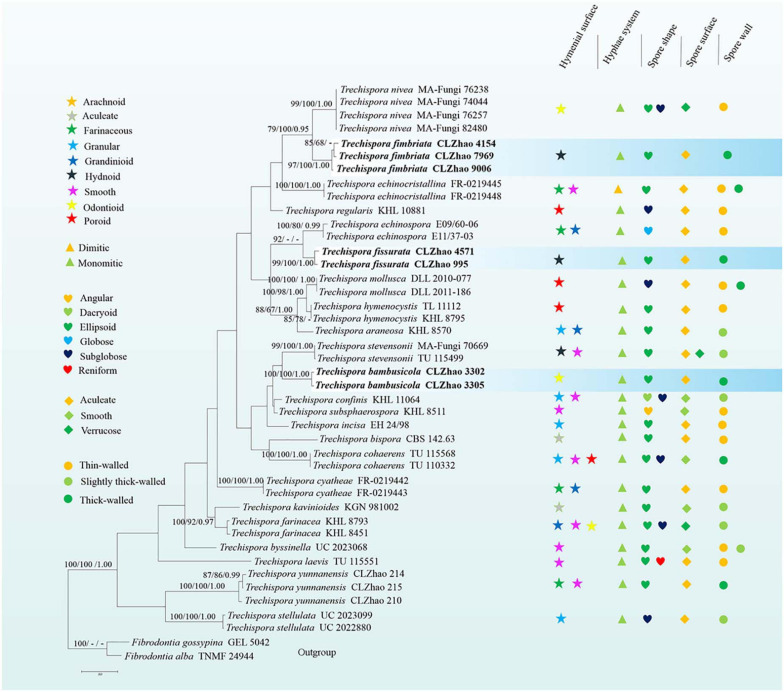
Maximum Parsimony strict consensus tree illustrating the phylogeny of three new species and related species in *Trechispora* based on ITS + nLSU sequences. Branches are labeled with maximum likelihood bootstrap values > 70%, parsimony bootstrap proportion values > 50%, and Bayesian posterior probabilities > 0.95, respectively.

The phylogenetic tree (Figure 2) inferred from ITS + nLSU sequences demonstrated 27 species of *Trechispora* and revealed that *T. bambusicola* formed a single lineage and then grouped with *Trechispora rigida* (Berk.) K.H. Larss. and *T. stevensonii*. *T. fimbriata* was sister to *T. nivea*. *T. fissurata* grouped with *T. thelephora* (Lév.) Ryvarden.

### Taxonomy

#### *Trechispora bambusicola* C.L. Zhao, sp. nov.

**FIGURE 3:**
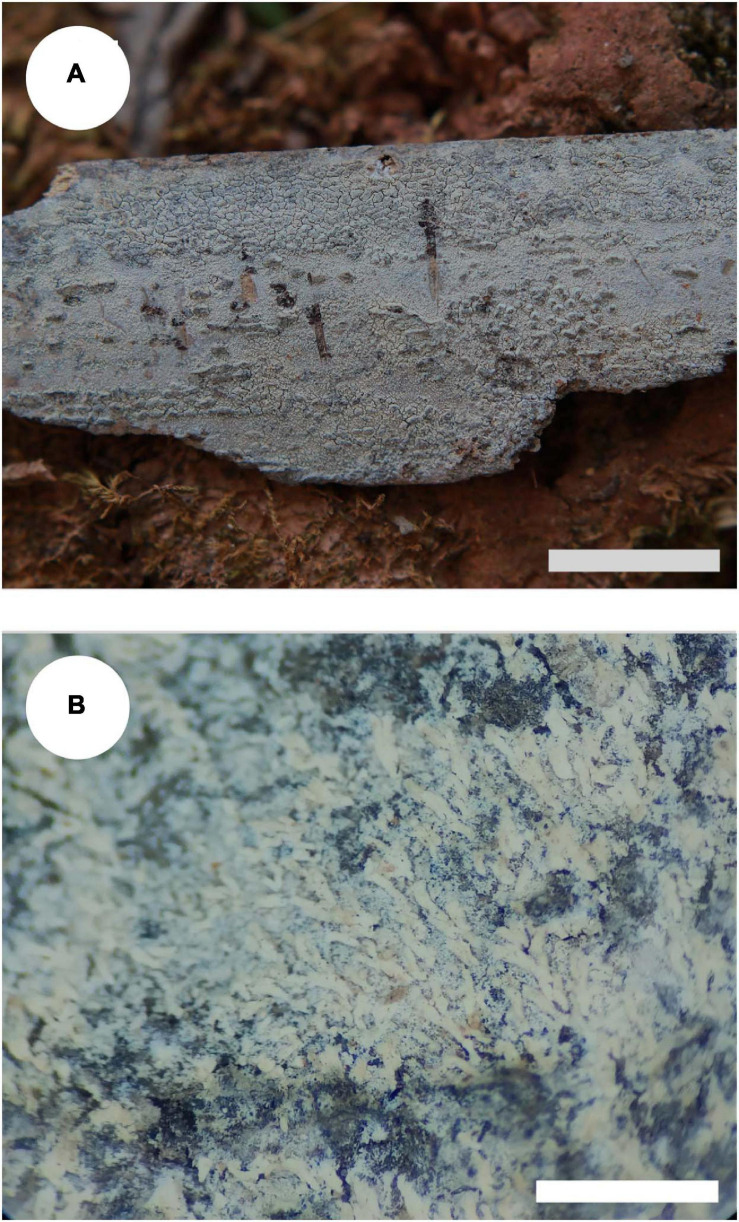
Basidiomata of *Trechispora bambusicola* (holotype): Bars: **(A)** 2 cm; **(B)** 1 mm.

**FIGURE 4:**
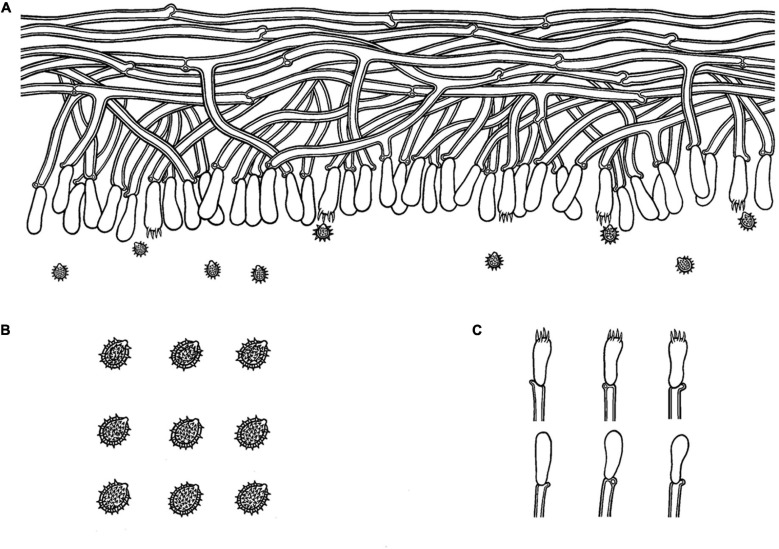
Microscopic structures of *Trechispora bambusicola* (drawn from the holotype): **(A)** Section of hymenium. **(B)** Basidiospores. **(C)** Basidia and basidioles. Bars: **(A,C)** 10 μm; **(B)** 5 μm.

*MycoBank no*.: MB 838612 (Figures 3, 4).

Holotype—China, Yunnan Province, Pu’er, Laiyanghe National Forest Park, on dead bamboo, 30 September 2017, CLZhao 3305 (SWFC).

Etymology—*Bambusicola* (Lat.): referring to occurrence on bamboo stump.

##### Basidiomata

Annual, adnate, soft, and fragile, without odor or taste when fresh, becoming granulose upon drying, up to 15 cm long and 5 cm wide, 50–300 μm thick. Hymenial surface odontioid, aculei cylindrical to conical, blunt, 0.3–0.5 mm long, white to cream when fresh, turn to cream to buff upon drying. Margin white to cream.

##### Hyphal structure

Monomitic, generative hyphae with clamp connections, hyaline, thick-walled, up to 0.7 μm, richly branched, 2–3 μm in diameter, IKI−, CB−; hyphae unchanged in KOH.

##### Hymenium

Cystidia and cystidioles absent; basidia shortly cylindrical to subclavate with median constriction, with 4-sterigmata and a basal clamp connection, 9–13 × 2.5–5 μm, basidioles dominant, in shape similar to basidia, but slightly smaller.

##### Basidiospores

Ellipsoid, hyaline, thick-walled, ornamented, sparse aculei, sharp, IKI−, CB−, (2.6−)2.9–3.5(−3.9) × 2–2.7 μm, *L* = 3.18 μm, *W* = 2.41 μm, *Q* = 1.26–1.38 (*n* = 60/2).

##### Type of rot

White rot.

##### Additional specimen examined

CHINA, Yunnan Province, Pu’er, Laiyanghe National Forestry Park, on dead bamboo, 30 September 2017, CLZhao 3302 (SWFC).

#### *Trechispora fimbriata* C.L. Zhao, sp. nov.

**FIGURE 5:**
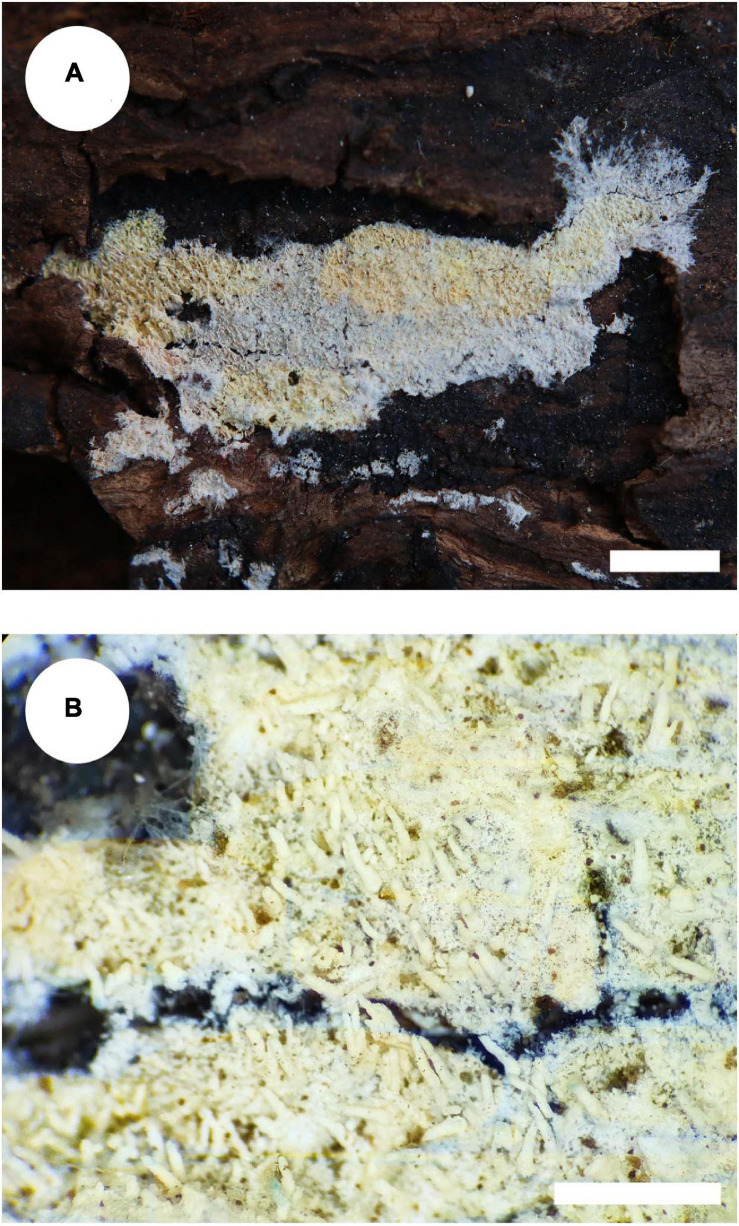
Basidiomata of *Trechispora fimbriata* (holotype). Bars: **(A)** 5 mm; **(B)** 1 mm.

**FIGURE 6:**
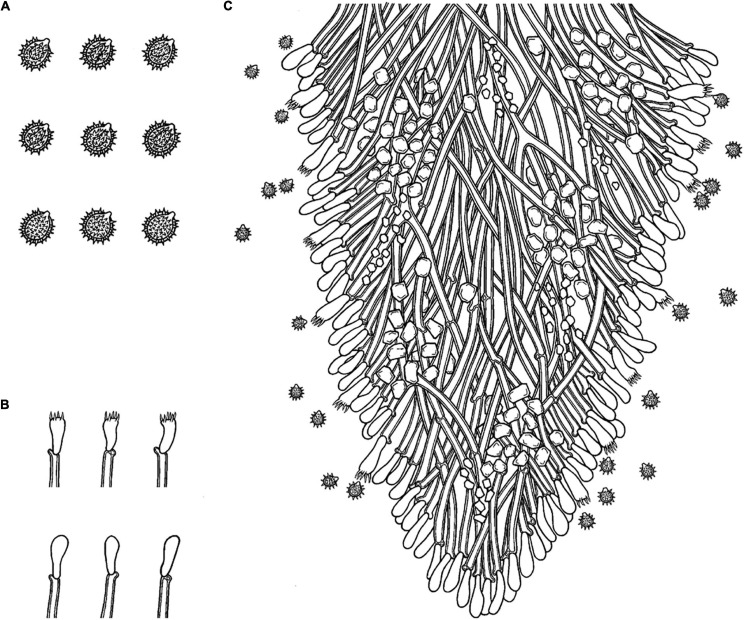
Microscopic structures of *Trechispora fimbriata* (drawn from the holotype). **(A)** Basidiospores. **(B)** Basidia and basidioles. **(C)** Section of hymenium. Bars: **(A)** 5 μm; **(B,C)** 10 μm.

*MycoBank no*.: MB 838613 (Figures 5, 6).

Holotype—China, Yunnan Province, Puer, Jingdong County, Wuliangshan National Nature Reserve, on the angiosperm trunk, October 5, 2017, CLZhao 4154 (SWFC).

Etymology—*Fimbriata* (Lat.): refers to the fimbriate margin of the basidiomata.

##### Basidiomata

Annual, adnate, without odor or taste when fresh, becoming fragile upon drying, up to 10 cm long and 3 cm wide, 100–200 μm thick. Hymenial surface hydnoid, with aculei, cylindrical, blunt, 0.4–0.7 mm long, white to pink when fresh, turn to pink to buff upon drying. Margin white to cream, thinning out, fimbriate.

##### Hyphal system

Monomitic, generative hyphae with clamp connections, hyaline, thick-walled, up to 0.6 μm, branched, 2–4 μm in diameter, IKI−, CB−; hyphae unchanged in KOH.

##### Hymenium

Cystidia and cystidioles absent; basidia shortly cylindrical with median constriction, with 4–6 sterigmata and a basal clamp connection, 7–11.5 × 3.5–5 μm, basidioles dominant, in shape similar to basidia, but slightly smaller.

##### Basidiospores

Ellipsoid, hyaline, thick-walled, ornamented, sparse aculei, sharp, IKI−, CB−, (2.5−)3–3.6(−3.8) × 2.4–3.2 μm, *L* = 3.25 μm, *W* = 2.63 μm, *Q* = 1.17–1.38 (*n* = 90/3).

##### Type of rot

White rot.

##### Additional specimens examined

China, Yunnan Province, Yuxi, Xinping County, Mopanshan National Forestry Park, on living tree of angiosperm, August 9, 2018, CLZhao 7969 (SWFC); on angiosperm trunk, October 15, 2018, CLZhao 9006 (SWFC).

#### *Trechispora fissurata* C.L. Zhao, sp. nov.

**FIGURE 7:**
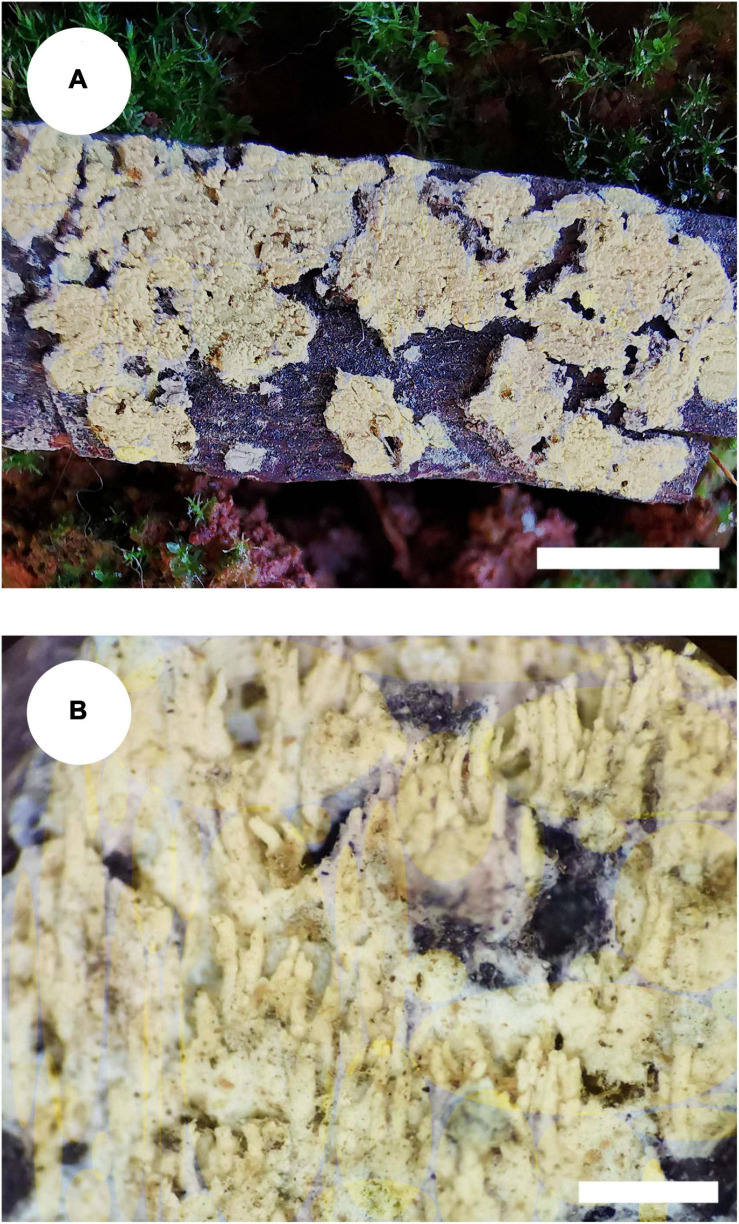
Basidiomata of *Trechispora fissurata* (holotype). Bars: **(A)** 1 cm; **(B)** 1 mm.

**FIGURE 8:**
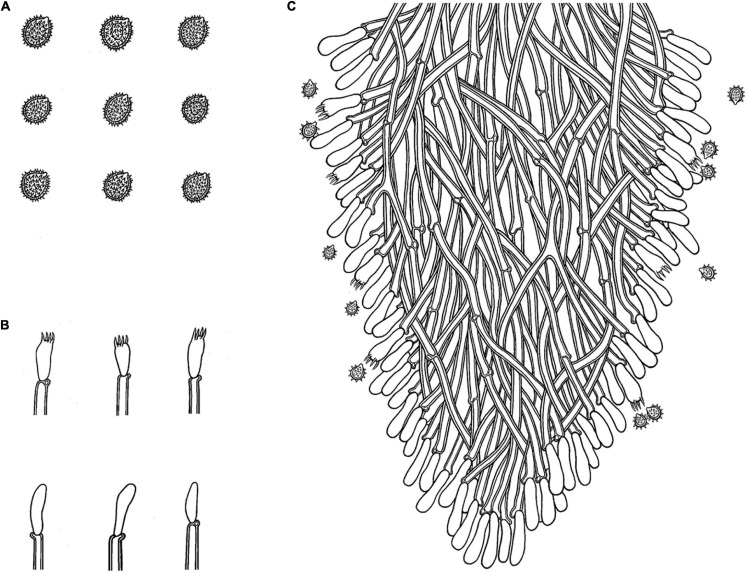
Microscopic structures of *Trechispora fissurata* (drawn from the holotype). **(A)** Basidiospores. **(B)** Basidia and basidioles. **(C)** Section of hymenium. Bars: **(A)** 5 μm; **(B,C)** 10 μm.

*MycoBank no*.: MB 838614 (Figures 7, 8).

Holotype—China, Yunnan Province, Puer, Jingdong County, Wuliangshan National Nature Reserve, on angiosperm trunk, October 6, 2017, CLZhao 4571 (SWFC).

Etymology—*Fissurata* (Lat.): refers to the cracking fissures on hymenial surface.

##### Basidiomata

Annual, adnate, without odor or taste when fresh, becoming cracking upon drying, up to 8 cm long and 4.5 cm wide, 400–800 μm thick. Hymenial surface hydnoid, with aculei, cylindrical to conical, sharp, 0.5–0.9 mm long, cream to straw yellow when fresh, turn to cream to yellow upon drying. Margin cream to yellow.

##### Hyphal system

Monomitic, generative hyphae with clamp connections, hyaline, thick-walled, up to 0.8 μm, branched, 2.5–5 μm in diameter, IKI−, CB−; hyphae unchanged in KOH.

##### Hymenium

Cystidia and cystidioles absent; basidia shortly clavate to tubular, with 4-sterigmata and a basal clamp connection, 8–10.5 × 2.5–4.5 μm, basidioles dominant, in shape similar to basidia, but slightly smaller.

##### Basidiospores

Ellipsoid, hyaline, thick-walled, ornamented, dense aculei, sharp, IKI−, CB−, (3−)3.3–4(−4.3) × (2.5−)2.8–3.5(−3.9) μm, *L* = 3.67 μm, *W* = 3.19 μm, *Q* = 1.13–1.17 (*n* = 60/2).

##### Type of rot

White rot.

##### Additional specimen examined

CHINA, Yunnan Province, Yuxi, Xinping County, Mopanshan Forestry Park, on the fallen angiosperm branch, January 17, 2017, CLZhao 995 (SWFC).

## Discussion

Phylogenetically, Phookamsak et al. (2019) introduced the phylogram generated from BI analysis of ITS sequence dataset of *Trechispora* sequences and included most taxa in this genus, in which it implied the phylogenetic relationship among species of *Trechispora*. In the present study, based on the ITS sequences (Figure 1), *T. bambusicola* was sister to *T. stevensonii* (Berk. and Broome) K.H. Larss; *T. fimbriata* grouped with *T. nivea*; *T. fissurata* grouped with *T. echinospora* Telleria, M. Dueñas, I. Melo, and M.P. Martín. Further ITS + nLSU dataset (Figure 2) revealed that *T. bambusicola* formed a single lineage and then grouped with *T. rigida* and *T. stevensonii*; *T. fimbriata* was sister to *T. nivea*; *T. fissurata* grouped with *T. thelephora*. However, *T. rigida* differs in its dirty white to buff hymenophore (Larsson, 1996). *T. stevensonii* is separated from *T. bambusicola* by the smooth to hydnoid hymenophore and larger basidiospores (4–4.5 × 3–3.5 μm; Larsson, 1995). *T. nivea* differs from *T. fimbriata* by the white to light ochraceous hymenial surface (Persoon, 1794). *T. echinospora* differs from *T. fissurata* by the farinaceous to grandinioid hymenophore and larger, globose basidiospores (3.3–4 × 2.8–3.5 μm; Phookamsak et al., 2019) and *T. thelephora* differs in its pileate to stipitate with light yellow brown surface and larger (4–5 × 3.4–4.5 μm; Albee-Scott and Kropp, 2010).

In the present study, three new species, *T. bambusicola*, *T. fimbriata*, and *T. fissurata* spp. nov. are found from rotten wood. Morphologically, *T. bambusicola* is similar to *T. cyatheae* Ordynets, Langer and K.H. Larss. by sharing the characteristics of soft and fragile basidiomata. However, *T. cyatheae* differs from *T. bambusicola* by having farinaceous to grandinioid hymenophore and thin-walled generative hyphae (Ordynets et al., 2015).

*Trechispora fimbriata* has similar characteristics of having the fimbriate margin with *Trechispora canariensis* Ryvarden and Liberta, *Trechispora clancularis* (Park.-Rhodes) K.H. Larss., *Trechispora microspora* (P. Karst.) Liberta, *Trechispora stellulata* (Bourdot and Galzin) Liberta, and *Trechispora subhelvetica* (Parmasto) Liberta. However, *T. canariensis* differs in its arachnoid to pelliculose hymenophore and larger basidiospores (5–7 × 3–3.5 μm; Ryvarden and Liberta, 1978); *T. clancularis* differs in the poroid to irpicoid hymenophore and slightly cyanophilous basidiospores (Larsson, 1994); *T. stellulata* differs in the arachnoid to byssoid hymenophore with whitish hymenial surface (Liberta, 1966); and *T. subhelvetica* differs in the narrower basidiospores (3–4 × 2–2.5 μm; Parmasto, 1965).

*Trechispora fissurata* resembles several species with similar features of having the hydnoid hymenophore and a monomitic hyphal system: *T. nivea* (Pers.) K.H. Larss., *T. stevensonii* (Berk. and Broome) K.H. Larss., and *Trechispora verruculosa* (G. Cunn.) K.H. Larss., but *T. nivea* by the white to pale ochraceous hymenial surface and thin-walled generative hyphae encrusted with granular crystals (Bernicchia and Gorjón, 2010); *T. stevensonii* by the white to ochraceous hymenial surface and larger basidiospores (4–4.5 × 3–3.5 μm; Larsson, 1995); *T. verruculosa* by the slightly cyanophilous and larger basidiospores (4.5–5.5 × 3.5–4.5 μm; Larsson, 1996).

Currently, eight species of *Trechispora* have been reported from China (Dai, 2011; Xu et al., 2019), *Trechispora alnicola*, *Trechispora cohaerens*, *T. farinacea*, *T. microspora*, *T. nivea*, *Trechispora polygonospora* Ryvarden, *Trechispora subsphaerospora* (Litsch.) Liberta, and *T. yunnanensis*, and one species of *T. yunnanensis* was found in Yunnan Province of China and it differs from three new species by having a smooth to farinaceous hymenial surface and larger basidiospores (7–8.5 × 5–5.5 μm; Xu et al., 2019). Three new taxa do not closely group together in phylogenetic trees, and morphologically, *T. bambusicola* differs from *T. fimbriata* and *T. fissurata* by having granulose basidiomata with cream to buff hymenial surface and growth on dead bamboo. *T. fimbriata* differs in its fimbriate margin of the basidiomata with pink to buff hymenial surface.

In addition, the ectomycorrhizal fungi (EcM) play an important role in ecosystems based on their mutualistic association with many groups of plants (Heijden et al., 2015). Vanegas-León et al. (2019) discovered the Trechisporales basidiomes and root colonization from *T. thelephora* basidiome. In the present study, *T. fissurata* was sister to *T. thelephora* based on ITS + nLSU phylogenetic analysis (Figure 2), which implied that both species have close evolutionary relationship. However, *T. fissurata* grows on deeply decayed wood, and *T. thelephora* is a soil-inhabiting fungus. Therefore, future investigations in both inhabiting types are needed to determine whether the natural selection or other factors pushes the different direction on inhabiting soil/wood among *Trechispora*.

In the habitat and distribution, Hibbett et al. (2014) revealed that most species of *Trechispora* is considered as soil-inhabiting. Later, some species were found on deeply decayed wood fungi (Bernicchia and Gorjón, 2010; Dai, 2011). However, some species in *Trechispora* are a typical feature of ectomycorrhizal fungi as frequently forming basidiomes on soil (Dunham et al., 2007; Vanegas-León et al., 2019). In the neotropical and subtropical region, the ectomycorrhizal basidiomes are found; however, the researches on the new taxa related to wood-decaying fungi of *Trechispora* from China are poorly reported. Further studies may focus on the relationships between the plants and species from *Trechispora* and try to better understand the evolutionary directions between soil-inhabiting and decayed wood fungi of *Trechispora*; many fungal studies on phylogeny and application were from these areas, which will be useful to push future researches for the genus *Trechispora* (Dai, 2011; Cui et al., 2019; Shen et al., 2019; Zhu et al., 2019; Richter et al., 2019; Angelini et al., 2020; Bao et al., 2020).

## Disclosure

All the experiments undertaken in this study comply with the current laws of the People’s Republic of China.

## Data Availability Statement

The data presented in the study are deposited in the https://www.ncbi.nlm.nih.gov/GenBank and https://www.mycobank.org/page/Home/MycoBank repository accession number of GenBank (ITS MW544021-MW544027 and nLSU MW520171-MW520177) and MycoBank (MB 838612-MB 838614).

## Author Contributions

C-LZ collected the species. WZ performed the molecular phylogenetic analyses. Both authors were responsible for the morphological analysis and description of the collections, planned, organized, and evaluated critically the experimental parts, wrote the manuscript, contributed to the article, and approved the submitted version.

## Conflict of Interest

The authors declare that the research was conducted in the absence of any commercial or financial relationships that could be construed as a potential conflict of interest.

## Footnotes

1 http://www.indexfungorum.org/Names/Names.asp

2 https://www.mycobank.org/Biolomics.aspx?Table=Mycobank&Page=200&ViewMode=Basic

3 http://lutzonilab.org/nuclear-ribosomal-dna/

4 http://mafft.cbrc.jp/alignment/server/

5 www.phylo.org

## Abbreviations

ITS: internal transcribed spacer
nLSU: large subunit
SWFC: herbarium of Southwest Forestry University Kunming China
KOH: 5% potassium hydroxide
CB: Cotton Blue
CB −: acyanophilous
IKI: Melzer’s reagent
IKI −: both inamyloid and indextrinoid
L: mean spore length (arithmetic average for all spores)
W: mean spore width (arithmetic average for all spores)
Q: variation in the L/W ratios between The studied specimensn (a/b), number of spores (a) measured from given number (b) of specimens spore measurements do not include ornamentation
CTAB: cetyltrimethylammonium bromide
DNA: deoxyribonucleic acid
PCR: polymerase chain reaction
MP: maximum parsimony
ML: maximum likelihood
BI: Bayesian inference
TBR: tree-bisection reconnection.

## References

[B1] Albee-Scott S. Kropp B. R. (2010). A phylogenetic study of *Trechispora thelephora*. *Mycotaxon* 114 395–399. 10.5248/114.395 30528588

[B2] Angelini C. Vizzini A. Justo A. Bizzi A. Kaya E. (2020). First report of a neotropical agaric (*lepiota spiculata*, agaricales, basidiomycota) containing lethal α-amanitin at toxicologically relevant levels. *Front. Microbiol.* 11:1833. 10.3389/fmicb.2020.01833 32849433 PMC7432468

[B3] Bao D. F. Mckenzie E. H. C. Bhat D. J. Hyde K. D. Su H. Y. (2020). *Acrogenospora* (acrogenosporaceae, minutisphaerales) appears to be a very diverse genus. *Front. Microbiol.* 11:1606. 10.3389/fmicb.2020.01606 32793142 PMC7393737

[B4] Bernicchia A. Gorjón S. P. (2010). *Fungi Europaei 12: Corticiaceae s.l.* Alassio: Edizioni Candusso.

[B5] Cui B. K. Li H. J. Ji X. Zhou J. L. Song J. Si J. (2019). Species diversity, taxonomy and phylogeny of Polyporaceae (Basidiomycota) in China. *Fungal Divers.* 97 137–392. 10.1007/s13225-019-00427-4

[B6] Dai Y. C. (2011). A revised checklist of corticioid and hydnoid fungi in China for 2010. *Mycoscience* 52 69–79. 10.1007/S10267-010-0068-1

[B7] Dai Y. C. (2012). Polypore diversity in China with an annotated checklist of Chinese polypores. *Mycoscience* 53 49–80. 10.1007/s10267-011-0134-3

[B8] Dunham S. M. Larsson K. H. Spatafora J. W. (2007). Species richness and community composition of mat-forming ectomycorrhizal fungi in old-and second-growth Douglas-fir forests of the Hj Andrews Experimental Forest, Oregon, USA. *Mycorrhiza* 17 633–645. 10.1007/s00572-007-0141-6 17638027

[B9] Felsenstein J. (1985). Confidence intervals on phylogenetics: an approach using bootstrap. *Evolution* 39 783–791. 10.1111/j.1558-5646.1985.tb00420.x 28561359

[B10] Hall T. A. (1999). BioEdit: a user-friendly biological sequence alignment editor and analysis program for Windows 95/98/NT. *Nucleic Acids Symp. Ser.* 41 95–98. 10.1021/bk-1999-0734.ch008

[B11] Heijden M. G. Martin F. M. Selosse M. A. Sanders I. R. (2015). Mycorrhizal ecology and evolution: the past, the present, and the future. *New Phytol.* 205 1406–1423. 10.1111/nph.13288 25639293

[B12] Hibbett D. S. Bauer R. Binder M. Giachini A. J. Hosaka K. Justo A. (2014). “14 Agaricomycetes,” in *Systematics and evolution*, eds McLaughlin D. J. Spatafora J. W. (Berlin: Springer), 373–429.

[B13] Karsten P. A. (1890). Fragmenta mycologica XXIX. *Dova Hedwig.* 29 147–149.

[B14] Larsson K. H. (1994). Poroid species in *Trechispora* and the use of calcium oxalate crystals for species identification. *Mycol. Res.* 98 1153–1172. 10.1016/S0953-7562(09)80200-1

[B15] Larsson K. H. (1995). Taxonomy of *Trechispora farinacea* and proposed synonyms I. Species with a grandinioid or hydnoid hymenophore. *Symb. Bot. Ups.* 30 101–118.

[B16] Larsson K. H. (1996). New species and combinations in *Trechispora* (Corticiaceae, Basidiomycotina). *Nord. J. Bot.* 16 83–98. 10.1111/j.1756-1051.1996.tb00218.x

[B17] Larsson K. H. (2007). Re-thinking the classification of corticioid fungi. *Mycol. Res.* 111 1040–1063. 10.1016/j.mycres.2007.08.001 17981020

[B18] Larsson K. H. Larsson E. Koljalg U. (2004). High phylogenetic diversity among corticioid homobasidiomycetes. *Mycol. Res.* 108 983–1002. 10.1017/S0953756204000851 15506012

[B19] Liberta A. E. (1966). On *Trechispora*. *Taxon* 15 317–319. 10.2307/1216118

[B20] Liberta A. E. (1973). The genus *Trechispora* (Basidiomycetes, Corticiaceae). *Canad. J. Bot.* 51 1871–1892. 10.1139/b73-240

[B21] Liu S. L. Ma H. X. He S. H. Dai Y. C. (2019). Four new corticioid species in Trechisporales (Basidi omycota) from East Asia and notes on phylogeny of the order. *MycoKeys* 48 97–113. 10.3897/mycokeys.48.31956 30930653 PMC6420480

[B22] Miettinen O. Larsson K. H. (2006). *Trechispora elongata* species nova from North Europe. *Mycotaxon* 96 193–198.

[B23] Miller M. A. Holder M. T. Vos R. Midford P. E. Liebowitz T. Chan L. (2009). *The CIPRES Portals. – CIPRES*. Available online at: http://www.phylo.org/sub_sections/portal (accessed December 7, 2011).

[B24] Nylander J. A. A. (2004). *MrModeltest v2. Program distributed by the author.* Uppsala: Evolutionary Biology Centre.

[B25] Ordynets A. Larsson K. H. Langer E. (2015). Two new *Trechispora* species from La Réunion Island. *Mycol. Progr.* 14:113. 10.1007/s11557-015-1133-0

[B26] Parmasto E. (1965). Corticiaceae U. R. S. S. I. Descriptiones taxorum. Combinationes noval [novarum]. *Izv. Akad. Nauk Ėstonsk. SSR, Ser. Biol*. 14, 220–223.

[B27] Persoon C. H. (1794). Neuer Versuch einer systematischen Eintheilung der Schwämme. *Neues Mag. Bot.* 1 63–80.

[B28] Petersen J. H. (1996). *Farvekort*. *The Danish Mycological Society’s colour-chart.* Greve: Foreningen til Svampekundskabens Fremme.

[B29] Phookamsak R. Hyde K. D. Jeewon R. Bhat D. J. Jones E. B. G. Maharachchikumbura S. (2019). Fungal diversity notes 929-1035: taxonomic and phylogenetic contributions on genera and species of fungi. *Fungal Divers.* 95 1–273. 10.1007/s13225-019-00421-w

[B30] Richter C. Yurkov A. M. Boekhout T. Stadler M. (2019). Diversity of *Tilletiopsis*-Like Fungi in Exobasidiomycetes (Ustilaginomycotina) and Description of Six Novel Species. *Front. Microbiol.* 10:2544. 10.3389/fmicb.2019.02544 31824440 PMC6883903

[B31] Ronquist F. Huelsenbeck J. P. (2003). MrBayes 3: B2017ayesian phylogenetic inference under mixed models. *Bioinformatics* 19 1572–1574. 10.1093/bioinformatics/btg180 12912839

[B32] Ryvarden L. (2002). A note on the genus *Hydnodon* Banker. Some neotropical wood inhabiting fungi. *Syn. Fungorum* 15 31–33.

[B33] Ryvarden L. Liberta A. E. (1978). Contribution to the Aphyllophoralles of the Canary Islands 4. Two new species of *Trechispora* and *Xenmastella*. *Canad. J. Bot.* 56 2617–2619. 10.1139/b78-314

[B34] Shen L. L. Wang M. Zhou J. L. Xing J. H. Cui B. K. Dai Y. C. (2019). Taxonomy and phylogeny of *Postia*. Multi-gene phylogeny and taxonomy of the brown-rot fungi: *Postia and its related genera*. *Persoonia* 42 101–126. 10.3767/persoonia.2019.42.05 31551616 PMC6712536

[B35] Swofford D. L. (2002). *PAUP^∗^: Phylogenetic Analysis Using Parsimony (^∗^and Other Methods). Version 4.0b10.* Sunderland, MA: Sinauer Associates.

[B36] Telleria M. T. Melo I. Dueñas M. Larsson K. H. Paz Martin M. P. (2013). Molecular analyses confirm *Brevicellicium* in Trechisporales. *IMA Fungus* 4 21–28. 10.5598/imafungus.2013.04.01.03 23898409 PMC3719203

[B37] Trichiès G. Schultheis B. (2002). *Trechispora* antipus sp. nov., une seconde espèce bisporique du genre *Trechispora* (Basidiomycota, Stereales). *Mycotaxon* 82 453–458.

[B38] Vanegas-León M. L. Sulzbacher M. A. Rinaldi A. C. Mélanie Roy Neves M. A. (2019). Are trechisporales ectomycorrhizal or non-mycorrhizal root endophytes? *Mycol. Progr.* 18 1231–1240. 10.1007/s11557-019-01519-w

[B39] Vu D. Groenewald M. de Vries M. Gehrmann T. Stielow B. Eberhardt U. (2019). Large-scale generation and analysis of filamentous fungal DNA barcodes boosts coverage for kingdom fungi and reveals thresholds for fungal species and higher taxon delimitation. *Stud. Mycol.* 92 135–154. 10.1016/j.simyco.2018.05.001 29955203 PMC6020082

[B40] White T. J. Bruns T. Lee S. Taylor J. (1990). “Amplification and direct sequencing of fungal ribosomal RNA genes for phylogenetics,” in *PCR Protocols: A Guide to Methods And Applications*, eds Innis M. A. Gelfand D. H. Sninsky J. J. White T. J. (San Diego, CA: Academic Press), 315–322. 10.1016/B978-0-12-372180-8.50042-1

[B41] Xu T. M. Chen Y. H. Zhao C. L. (2019). *Trechispora yunnanensis* sp. nov. from China. *Phytotaxa* 424 253–261. 10.11646/phytotaxa.424.4.5

[B42] Yurchenko E. Wu S. H. (2014). *Fibrodontia alba* sp. nov. (Basidiomycota) from Taiwan. *Mycoscience* 55 336–343. 10.1016/j.myc.2013.12.004

[B43] Zhao C. L. Wu Z. Q. (2017). *Ceriporiopsis kunmingensis* sp. nov. (Polyporales, Basidiomycota) evidenced by morphological characters and phylogenetic analysis. *Mycol. Progr.* 16 93–100. 10.1007/s11557-016-1259-8

[B44] Zhu L. Song J. Zhou J. L. Si J. Cui B. K. (2019). Species diversity, phylogeny, divergence time and biogeography of the genus *Sanghuangporus* (Basidiomycota). *Front. Microbiol.* 10:812. 10.3389/fmicb.2019.00812 31057518 PMC6478708

